# Detection of a novel astrovirus from a black-naped monarch (*Hypothymis azurea)* in Cambodia

**DOI:** 10.1186/s12985-015-0413-2

**Published:** 2015-11-04

**Authors:** Ian H. Mendenhall, Katherine Nay Yaung, Priscilla H. Joyner, Lucy Keatts, Sophie Borthwick, Erica Sena Neves, Sorn San, Martin Gilbert, Gavin JD Smith

**Affiliations:** Program in Emerging Infectious Diseases, Duke-NUS Graduate Medical School, 8 College Rd, Singapore, 169857 Singapore; Wildlife Conservation Society, 2300 Southern Blvd, Bronx, NY 10460 USA; National Veterinary Research Institute, Phum Trea – Sarla Street (371) Sangkat, Phnom Penh, Cambodia; Present Address: Smithsonian Conservation Biology Institute, Front Royal, VA USA

**Keywords:** Avian astrovirus, Cambodia, Novel virus, Passerine

## Abstract

**Background:**

Astroviruses are comprised of two genera with *Avastrovirus* infecting birds and *Mamastrovirus* infecting mammals. Avastroviruses have primarily been associated with infections of poultry, especially chicken, turkey, duck, and guineafowl production systems, but also infect wading birds and doves. Outcomes result in a spectrum of disease, ranging from asymptomatic shedding to gastroenteritis with diarrhea, stunting, failure to thrive and death.

**Findings:**

Virological surveillance was conducted in birds from two sites in Cambodia in 2010. Samples were screened for influenza, astroviruses, coronaviruses, flaviviruses, and paramyxoviruses. A total of 199 birds were tested and an astrovirus was detected in a black-naped monarch (*Hypothymis azurea)*.

**Conclusions:**

This is the first astrovirus detection in a passerine bird. Phylogenetic analysis and nucleotide distances suggest that this avastrovirus forms a distinct lineage and may constitute a fourth avastrovirus group.

**Electronic supplementary material:**

The online version of this article (doi:10.1186/s12985-015-0413-2) contains supplementary material, which is available to authorized users.

## Findings

Astroviruses are comprised of two genera with *Avastrovirus* infecting birds and *Mamastrovirus* infecting mammals. These viruses are primarily transmitted fecal-orally, which is facilitated in agricultural systems due to host proximity, but they can persist in water [1]. Avastroviruses have primarily been associated with infections of poultry, especially chicken, turkey, duck, and guineafowl production systems. Outcomes result in a spectrum of disease, ranging from asymptomic shedding to gasteroenteritis with diarrhea, stunting, failure to thrive and death [2]. Phylogenetic analysis shows that *Avastrovirus* forms three major groups, with support for Group 1 avastroviruses (including chickens, guineafowl, and several duck species) forming a further three monophyletic clades [3].

Recently, avastroviruses have been discovered in a variety of wild birds, with the greatest numbers detected from aquatic species in the orders Anseriformes (i.e. teals, pintails, shovelers, and wigeons), Charadriiformes (i.e. greenshanks and sanderlings), and Pelecaniformes (i.e. herons and spoonbills) [3, 4]. The only land dwelling wild bird species found to harbor astroviruses include members of the order Colombiformes (i.e. doves and pigeons) and Coraciiformes (i.e. European roller) [5–7]. Aside from avian influenza virus, there have been few studies examining endemic viruses in birds in Cambodia. Here we report on the surveillance for a variety of viruses in species from four bird orders in Cambodia (Table 1) and the first detection of an astrovirus from the order Passeriformes.

**Table 1:** List of birds caught and sampled in Cambodia

Order	Species	Common Name	Jee Tour	Toul	Total
			(# AstV+)	Krasang	(# AstV+)
Anseriformes	*Anas poecilorhyncha*	Spot-billed duck	0	1	1
	*Dendrocygna javanica*	Lesser whistling-duck	0	3	3
Pelecaniformes	*Ardeola bacchus/speciosa*	Chinese/Javan pond heron	0	58	58
	*Egretta garzetta*	Little egret	0	1	1
	*Mesophoyx intermedia*	Intermediate egret	0	1	1
Gruiformes	*Gallicrex cinerea*	Watercock	0	10	10
Passeriformes	*Acrocephalus aedon*	Thick-billed warbler	2	0	2
	*Copsychus saularis*	Oriental magpie robin	5	0	5
	*Ficedula parva*	Red-throated flycatcher	1	0	1
	*Hypothymis azurea*	Black-naped monarch	2 (1)	0	2 (1)
	*Lanius cristatus*	Brown shrike	3	0	3
	*Lonchura punctulata*	Scaly-breasted munia	1	0	1
	*Lonchura striata*	White-rumped munia	1	0	1
	*Passer flaveolus*	Plain-backed sparrow	1	0	1
	*Pycnonotus blanfordi*	Streak-eared bulbul	7	0	7
	*Pycnonotus goiavier*	Yellow-vented bulbul	16	0	16
	*Rhipidura javanica*	Pied fantail	6	0	6
Total			45 (1)	74	119 (1)

From February until December 2010 the Wildlife Conservation Society collected samples from wild birds in Cambodia to study circulating viruses in the country’s avifauna. Birds were trapped at Toul Krasang, a wetland located in Kandal Province, and Jee Tour, a secondary forest in Takéo province under the University of Minnesota IACUC number 0702A02841. Paired oropharyngeal and cloacal swabs were collected from 119 birds at the two field sites (Table 1). Duplicate samples were taken and stored in either guanidine isothiocyanate or virus transport media for detection or culture, respectively. Samples were kept at −80 °C until shipped to Duke-NUS Graduate Medical School Singapore for PCR screening.

Paired samples in guanidine isothiocyanate were pooled, vortexed, centrifuged at 4,000 g for 5 min, and the clarified supernatant was removed for RNA extraction. Lysis buffer was added in a laminar flow hood before nucleic acid extraction using a QiaExtractor robot (Qiagen). Complementary DNA was synthesized using a Superscript II kit (Invitrogen) following the manufacturer’s protocol using either a Uni-12 specific primer for detection of influenza or with random hexamers for detection of the other virus families. A Taqman PCR assay was used to test for influenza A viruses, while family specific primer sets targeting conserved regions of the genome were used for detection of astroviruses, coronaviruses, flaviviruses, and paramyxoviruses (protocols and primer sets are available in Additional file 1: Supplementary Information).

An astrovirus positive PCR product from a black-naped monarch (*Hypothymis azurea*) was purified using a Qiagen PCR purification kit (Qiagen). This product was cloned using a Promega p-Gem T easy kit (ProMega). Plasmids were purified using an Omega MiniPrep (Omega) purification kit and sent for sequencing. Two sequences generated from the same individual in this study were deposited in GenBank (accession numbers KT965674-KT965975). Attempts to generate additional genetic data using a 3′ RACE PCR and culture in embryonated chicken eggs were unsuccessful.

The RNA dependent reverse polymerase (RdRp) sequences from representative mammal and bird species were aligned using MUSCLE in Geneious 7.1.6 [8] and then manually curated (see Additional file 2: Table S1). Nucleotide pairwise p-distances were calculated using Mega 6.06 [9]. Maximum-likelihood (ML) trees were constructed in Geneious 7.1.6 using PHYML v2.2.0 [10] using a combined NNI and SPR topology search and support calculated with 500 ML bootstrap replicates. Bayesian analysis was conducted in Geneious 7.1.6 with MrBayes v3.2.2 [11] using two replicates of 5,000,000 generations sampled every 1,000 generations. The convergence of chains and estimation of burn-in were assessed and Bayesian posterior probabilities were calculated from the consensus of 8,000 trees after excluding the first 2,000 trees as burn-in. Both analyses implemented a GTR + G nucleotide substitution model. Phylogenetic trees were visualized in FigTree v1.4.2 (http://tree.bio.ed.ac.uk/software/figtree/).

Astrovirus was detected in one black-naped monarch from 119 birds tested with an overall prevalence of 0.8 % (Table 1). Influenza viruses, coronaviruses, paramyxoviruses and flaviviruses were not detected. Comparison of the 391 bp nucleotide alignment of the two passerine avastrovirus RdRp clones detected in the black-naped monarch identified no polymorphic sites. For the remaining avastroviruses, pairwise nucleotide p-distance computations showed that within group similarity varied from 69.2 % (Group 1) to 77.6 % (Group 3). Between group nucleotide pairwise distances varied from 51.7 % (Group 2 vs Group 3) to 58.8 % similarity (Group 1 vs passerine avastrovirus) (Additional file 3: Table S2). These results suggest that the passerine avastrovirus is as divergent from the three described groups as those groups are from each other and may represent a unique avastrovirus lineage.

**Fig. 1:**
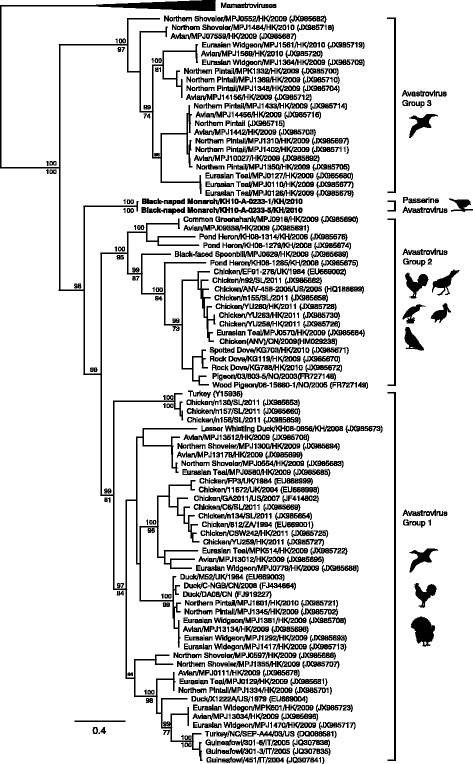
This tree represents a phylogenetic analysis on the RNA dependent reverse polymerase using Mr. Bayes and PHYML in Geneious 7.1.6. Posterior probability values (>95 %) from Mr. Bayes are above the nodes and Maximum Likelihood bootstrap values (>70 %) are labeled below the nodes. Sequences from this study are in red text. The sequences are labeled with the common name/sample identification/two letter country code/year collected (GenBank Accession). A list of all sequences is in the supplementary data

Both the ML and Bayesian analysis of the RdRp gene showed the same basic tree topology as Chu et al. [3] with the exception that the passerine astrovirus formed a divergent group that falls in a basal position to both avastrovirus groups 1 and 2 (Fig. 1). Groups 1 and 2 are strongly supported as sister groups to each other and are in turn most closely related to the black-naped monarch virus sequences. Group 3 avastroviruses are comprised entirely of ducks and supported as a sister clade to all other avastroviruses. Phylogenetic reconstruction and the pairwise distance analysis both indicate that the passerine avastrovirus forms a novel lineage that is well differentiated from all previously described avastroviruses.

This is the first astrovirus detection in a passerine bird. Phylogenetic analysis and nucleotide distances suggest that this avastrovirus forms a distinct lineage and may constitute a fourth avastrovirus group. Astroviruses are a genetically diverse group with a wide host range [12]. Avastroviruses have previously been detected from domestic birds that tend to be communally housed (chickens, ducks, turkeys), birds specifically associated with water bodies (ducks, herons, spoonbills, and shorebirds), or gregarious birds exhibiting communal behaviors (wood pigeons and rock doves). These spatial-temporal associations provide opportunities for transmission of viruses between receptive hosts [13]. However, it is unknown where and how this individual acquired the infection because although black-naped monarchs will forage in mixed flocks, they tend to be solitary or roost in pairs [14]. Interestingly, pond herons represented nearly 50 % of all birds sampled in our study, yet we detected no astrovirus positives even though avastroviruses have previously been detected in this species in Cambodia [3].

Our understanding of the impact of astrovirus infection in wild birds is very limited, especially regarding fitness costs and transmission dynamics. There is evidence that cross-species transmission occurs and that individual species may host divergent astrovirus strains, indicating their receptiveness to infection [15, 16]. Astroviruses can also undergo recombination leading to the emergence of novel strains [17]. Birds in the order Passeriformes are highly diverse, comprising 60 % of all bird species, and occupy a tremendous variety of terrestrial ecological niches [18]. As such, the discovery of an astrovirus in one species raises the possibility that additional astrovirus lineages may exist in the Passeriformes.

## Additional files

Additional file 1:
**Supplementary Information.** Protocols and primers used for screening bird samples. (PDF 74 kb) Additional file 2: Table S1. List of sequences used for phylogenetic tree. (XLSX 46 kb) Additional file 3: Table S2. Pairwise distance between avastrovirus groups. (XLSX 8 kb)
