# Supplementary material for: Detection of a novel astrovirus from a black-naped monarch (Hypothymis azurea) in Cambodia
Source: Virol J. 2015 Nov 4;12:182. doi: 10.1186/s12985-015-0413-2 (PMC4634723; doi:10.1186/s12985-015-0413-2)
Supplement: Additional file 1: — Supplementary Information. Protocols and primers used for screening bird samples. (PDF 74 kb) [file 12985_2015_413_MOESM1_ESM.pdf]

Supplementary Information. Protocols and primers used for screening bird samples

cDNA for influenza screening was synthesized using a Superscript II kit (Invitrogen) in a 20-µl reaction mixture following the manufacturer's protocol with 11.5-µl of RNA and a 10µM concentration of a Uni-12 specific primer (5'-AGCAAAAGCAGG-3'). A 5x RT Buffer, 0.1M dithiothreitol (DTT), 10mM deoxynucleoside triphosphate mix (dNTP), and 200U of SuperScript II RT. Samples were tested for influenza A using a Taqman qPCR assay designed to amplify a conserved region of the matrix gene. A 25-µl reaction was used containing 2x RT Buffer (Biorad), 10µM forward and reverse primer, 20µM probe, and 4-µl of cDNA (FluA-M-F 5'-CTTCTAACCGAGGTCGAAACGTA-3', FluA-M-R 5'-GGTGACAGGATTGGTCTTGTCTTTA-3', FluA-M-Probe 5'-56-FAM-TCAGGCCCCCTCAAAGCCGAC-3BHQ_1-3'). Reactions were run on a Biorad CFX96 Real Time System thermocycler. After an initial incubation at 95°C for 5 minutes, 45 cycles of amplification were carried out with a denaturation step at 95°C for 15 seconds and an annealing step at 60°C for 34 seconds (1).

Complementary DNA was also synthesized from RNA with random hexamers using a Superscript II kit (Invitrogen) following manufacturer's protocols. This cDNA was screened for astroviruses, coronaviruses, flaviviruses, and paramyxoviruses in an Applied Biosystems Veriti 96 well thermocycler. Astrovirus detection followed a heminested protocol where a 25-µl initial reaction was used containing 1x buffer (Thermo Scientific), 0.2mM dNTP mix, 0.5 µM F1, F2, and R primer (F1: 5'-GARTTYGATTGGRCKCGKTAYGA-3', F2: 5'-GARTTYGATTGGRCKAGGTAYGA-3', R: 5'-GGYTTKACCCACATNCCRAA-3', F1-hemi: 5'- CGKTAYGATGGKACKATHCC-3', F2-hemi: 5'-

AGGTAYGATGGKACKATHCC-3'), 1mM $MgCl_2$, and 1.25 units of Taq (Thermo Scientific). After an initial denaturation period at 94°C for 3 minutes, 30 cycles of amplification were carried out with a denaturation step at 94°C for 30 seconds, annealing at 50°C for 30 seconds, and extension at 68°C for 30 seconds. This was followed by a final extension at 68°C for 5 minutes before being held at 10°C until removal from the thermocycler. The heminested step used 1-μl of the first round PCR product as template and follows the same protocol as the first PCR (2).

Coronaviruses were tested with a PCR final volume of 25-μl containing a 1x buffer (Thermo Scientific), 0.2mM dNTP mix, 0.5 μM F and R primer (Corfor: 5'-GGTTGGGACTATCCTAAGTGTGA – 3', Correv: 5' - CCATCATCAGATAGAATCATCAT- 3'), 1mM $MgCl_2$, and 1.25 units of Taq (Thermo Scientific). cDNA synthesized from RNA with random hexamers was used as a template. After an initial denaturation period at 95°C for 2 minutes, 40 cycles of amplification were carried out with a denaturation step at 95°C for 15 seconds, annealing at 48°C for 30 seconds, and extension at 68°C for 1 minute. This was followed by a final extension at 68°C for 5 minutes before being held at 10°C until removal from the thermocycler (3).

Paramyxoviruses were tested using a heminested PCR protocol where a 25-μl initial reaction was used containing 1x buffer (Thermo Scientific), 0.2mM dNTP mix, 0.5 μM F1, F2, and R primer (ParaF1: 5'- GAAGGITATTGTCAIAARNTNTGGAC-3', ParaF2: 5'- GTTGCTTCAATGGTTCARGGNGAYAA-3', ParaR: 5'-GCTGAAGTTACIGGITCICCDATRTTNC -3'), 1mM $MgCl_2$, and 1.25 units of Taq (Thermo Scientific). cDNA synthesized from random hexamers were used as a template. After an initial denaturation period at 94°C for 2 minutes, 35 cycles of amplification were carried out with a denaturation step at 94°C for 15 seconds,

annealing at 49°C for 30 seconds, and extension at 72°C for 1 minute. This was followed by a final extension at 72°C for 5 minutes before being held at 10°C until removal from the thermocycler. The heminested step used 1-µl of the first round PCR product and follows the same protocol as the first PCR (4).

Flaviviruses were screened for using a PCR with a final volume of 25-µl containing a 1x buffer (Thermo Scientific), 0.2mM dNTP mix, 0.5 µM F and R primer (VIR2217F: 5'- TGTGTCTACAACATGATGGGAAAGAGAGA–3', VIR2217F: 5'- TGCTCCCAGCCACATGTACCA-3'), 1mM $MgCl_2$, and 1.25 units of Taq (Thermo Scientific). cDNA synthesized from random hexamers were used as a template. After an initial denaturation period at 95°C for 2 minutes, there were 8 cycles of 95°C for 30 seconds, 48°C for 30 seconds, and 72°C for 30 seconds, followed by 37 cycles of 95°C for 15 seconds, 56°C for 20 seconds, and 72°C for 20 seconds culminating with a 2 minute extension at 72°C and held at 10°C until processing (5).

References.

1. Whiley DM, Sloots TP. A 5'-nuclease real-time reverse transcriptase-polymerase chain reaction assay for the detection of a broad range of influenza A subtypes, including H5N1. Diagn Microbiol Infect Dis. 2005 Dec;53(4):335-7.

2. Chu DK, Poon LL, Guan Y, Peiris JS. Novel astroviruses in insectivorous bats. Journal of Virology. 2008 Sep;82(18):9107-14.

3. Vijgen L, Moes E, Keyaerts E, Li S, Van Ranst M. A pancoronavirus RT-PCR assay for detection of all known coronaviruses. Methods in Molecular Biology (Clifton, NJ). 2008;454:3-12.

4. Schatzberg SJ, Li Q, Porter BF, Barber RM, Claiborne MK, Levine JM, et al. Broadly reactive pan-paramyxovirus reverse transcription polymerase chain reaction and sequence analysis for the detection of Canine distemper virus in a case of canine meningoencephalitis of unknown etiology. Journal of Veterinary Diagnostic Investigation: Official publication of the American Association of Veterinary Laboratory Diagnosticians, Inc. 2009 Nov;21(6):844-9.

5. Grant-Klein RJ, Baldwin CD, Turell MJ, Rossi CA, Li F, Lovari R, et al. Rapid identification of vector-borne flaviviruses by mass spectrometry. Molecular and Cellular Probes. 2010 Aug;24(4):219-28.
